# The need for equity in interventional cardiology care in sub-Saharan Africa

**DOI:** 10.3389/fcvm.2026.1812816

**Published:** 2026-05-12

**Authors:** Ramon Moronkola, Abdulhammed Babatunde, Oluwaseye Oladimeji, Mesoma Okeke, Esther Opeyemi Ijaola, Alaba Busola Oladimeji, Folashade Daniel, Adeola Ajibare, Adebowale Adekoya, Obinnaya Emerole

**Affiliations:** 1 Cardiology Unit, Department of Medicine, Lagos State University Teaching Hospital, Ikeja, Lagos State, Nigeria; 2 Cardiology Unit, Department of Medicine, College of Medicine, University of Ibadan, Ibadan, Nigeria; 3 Cardiology Unit, Department of Medicine, Cardiovascular Education Foundation, Macon, GA, United States

**Keywords:** cardiology, CVDs, interventional cardiology, LMICs, Nigeria

## Abstract

There is a growing burden of cardiovascular diseases (CVDs) in sub-Saharan Africa (SSA). CVDs such as coronary artery disease, valvular heart disease, heart block and others require procedures involving interventional cardiology. However, there is a huge unmet need for catheterization labs and expertise in health facilities in SSA. We highlight the gaps in equitable access, service delivery and the urgent need for strategic collaborations to increase equitable access to interventional cardiology in the content. This review will guide policy making and inspire innovation in SSA to bridge the existing gap in CVDs management.

## 1 Introduction

In the past decade, the global burden of cardiovascular diseases has unfailingly risen, from being responsible for over 17 million deaths in 2013, to nearly 20 million in 2019 (1, 2). The increase in cardiovascular risk factors and lifestyle choices influence these rates beyond an overall global population rise (3). In sub-Saharan African countries such as Nigeria, there is limited resources for adequate and evidence-based management of the more prevalent cardiovascular diseases. Poverty and crowded living conditions in some parts further drive up the rate of conditions such as Rheumatic heart disease, the prevalence of which has otherwise declined in more developed regions (4). As a direct result or a sequela of the pathogenesis of these cardiovascular diseases, many conditions arise which require the need for interventional procedures. Interventional cardiology is a field of medicine that has rapidly evolved to meet this demand, showing up to improve diagnostic examinations and abate vascular conditions such as coronary atherosclerosis, valvular diseases, and other structural abnormalities (5, 6). These procedures offer a non-surgical invasive approach to relieving a range of cardiac conditions (7). Coronary artery and similar vascular diseases, which form highest percentage of conditions for which intervention cardiology is required, are on the rise globally (8). These group of diseases represent steps in the final common pathway of many cardiovascular conditions and risk factors such and hypertension, hyperlipidaemia, smoking, diet, sedentary lifestyle, and alcohol consumption, all of which are becoming increasingly prevalent (9).

While interventional cardiology services exist in health facilities in sub-Saharan Africa (SSA), mostly urban centers, there is a significant gap in providing comprehensive care for the broader population (10). Despite the rising burden of cardiovascular diseases on the continent, the infrastructure for advanced interventions lags (2). This disparity highlights a critical need for expanding access to coronary procedures, valvular interventions, and other specialized interventional procedures in SSA. The aim of this commentary is to address the realities of interventional cardiology in SSA, focusing on the gaps in equitable access, service delivery and the urgent need for strategic collaborations.

## 2 Existing system in sub-Saharan Africa

The landscape of existing systems providing interventional cardiology in SSA is faced with challenges. Across SSA, the existing systems are characterized by fragmented infrastructure, shortage of trained specialists, and dilemma of low patient load secondary to lack of access. Table 1 summarises the disparity and current state of interventional capacity across nine SSA countries with some published data. Cath lab density remains critically low across regions, Uganda and Cote d’Ivoire each have a single functional laboratory serving 48 and 27 million people respectively. In every country represented, out-of-pocket payment supplemented by philanthropic cardiac missions were the major financing mechanism with little commitment from government.

**Table 1:** A table that summarizing the available data on catheterization laboratory capacity, interventional cardiologist density, annual PCI volume, and funding mechanisms across sub-Saharan African countries.

Country	Population (millions)	No. of Cath Laboratories	Cath Labs per 1,000,000 Population	No. of Interventional Cardiologists (approx.)	Annual PCI Volume	Predominant Funding Mechanism(s)	References
South Africa	∼62	78 (all provinces) 12 (sub-Saharan provinces only)	∼1.3 (provincial) ∼0.2 (SSA provinces)	163 total cardiologists (fraction are interventional)	∼59.7% of STEMI patients received PCI (ACCESS SA registry)	Medical insurance (private) Out-of-pocket Limited public funding	(11)
Nigeria	∼213	∼24 (About 16 functional)	∼0.11	>25 interventional cardiologists (exact number undocumented)	∼350 PCIs/year (national estimate)	Out-of-pocket (majority) Philanthropic cardiac missions Limited private insurance Occasional government sponsorship	(12, 13)
Kenya	∼55	>6 (as of 2012; likely increased)	<0.1	Limited; exact number not published	Primary PCI rate ∼13% in STEMI (annual absolute volume not published)	Out-of-pocket Private insurance Limited public funding	(14)
Uganda	∼48	1 (Uganda Heart Institute, Kampala)	∼0.02	5 adult + 2 paediatric interventional cardiologists	3,542 total invasive procedures over 8 years (2012–2019) (∼443 procedures/year all types; peak of 30 PCIs in 2018)	Government (50% co-pay) International philanthropic organisations Out-of-pocket (50% co-pay)	(14)
Tanzania	∼63	≥2 (JKCI, Dar es Salaam; Benjamin Mkapa Hospital, Dodoma)	∼0.03	Small number; exact count not published	∼1,200 catheterisation procedures at JKCI in 2020 (∼10% were PCI ≈120 PCIs/year at JKCI alone)	Out-of-pocket High-premium private insurance NGO/philanthropic support (Madaktari Africa)	(15, 16)
Côte d'Ivoire (Ivory Coast)	∼27	1 (Abidjan Heart Institute – 24 h/7d service)	∼0.04	Small number; exact count not published	166 primary PCIs over 10 years (2010–2019) = ∼16–17 PCIs/year; Total STEMI PCI rate 34.9% (272/780 patients)	Out-of-pocket Philanthropic missions	(17)
Sudan	∼45	4 operational (all outside Khartoum due to conflict)	∼0.09	Limited; exact number not published post-conflict	100 PCIs documented at one cenetre over 1 year (2023–2024) during active conflict	Government: free thrombolytics and complementary cath lab access (policy, not always operative) Out-of-pocket	(18)
Cameroon	∼28	1 (Shisong Cardiac Centre – private)	∼0.04	Very limited	Not published	International philanthropic (private centre) Out-of-pocket	(19)
Senegal	∼18	Not formally published; limited capacity in Dakar	N/A	Very limited; cath ablation available (simple EP procedures only)	Not published	Out-of-pocket Philanthropic missions	(20)

Cath lab counts and cardiologist numbers reflect best available published figures and may underestimate true current capacity. PCI, percutaneous coronary intervention; IC, interventional cardiology; ACS, acute coronary syndrome; STEMI, ST-elevation myocardial infarction; SSA, sub-Saharan Africa; NGO, non-governmental organisation; JKCI, Jakaya Kikwete Cardiac Institute (Tanzania); UHI, Uganda Heart Institute. Countries excluded due to absence of any published quantitative data.

## 3 Infrastructure

**Figure 1:**
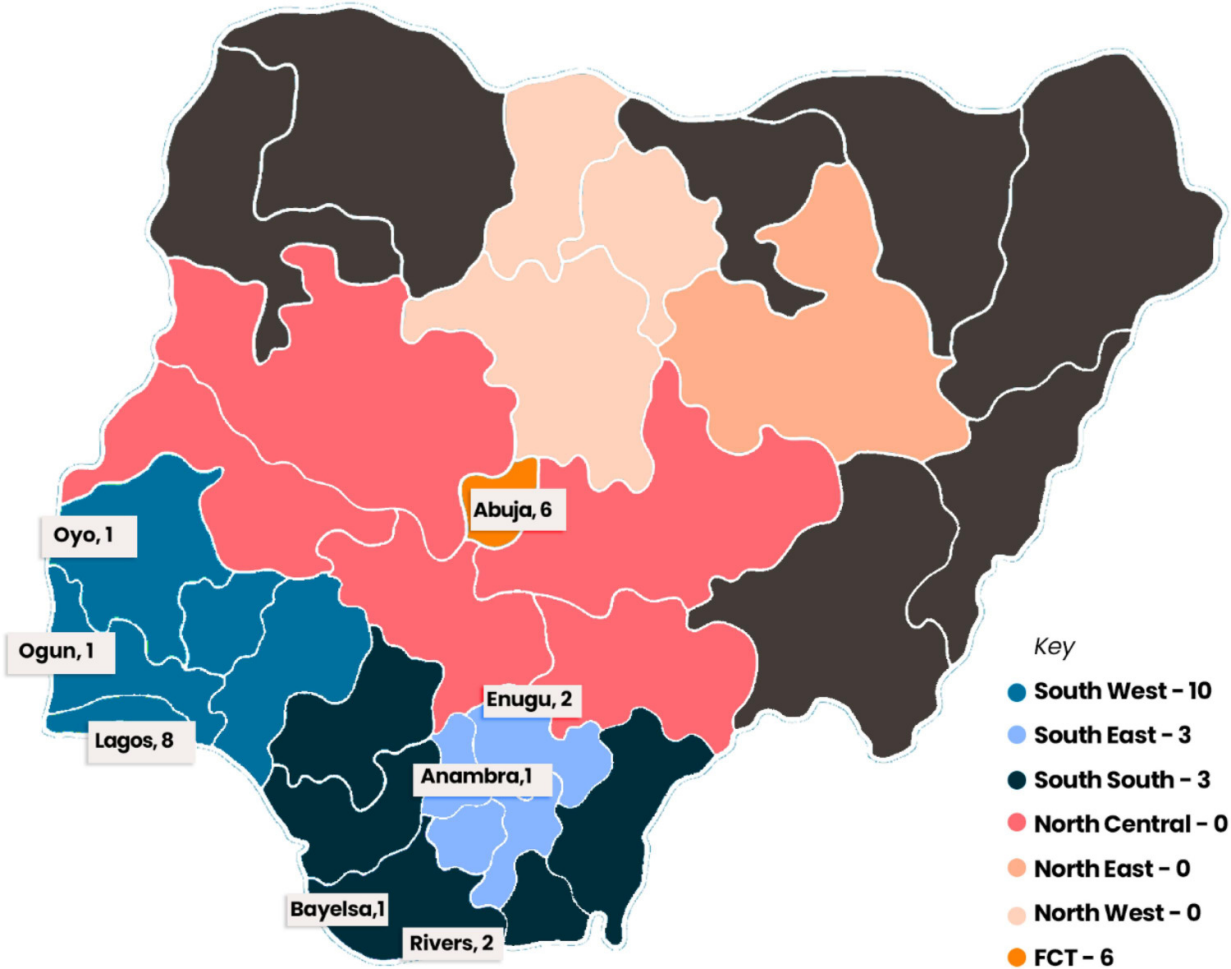
Figure showing the distribution of centers offering interventional cardiology across Nigeria. The list include the functional and non-functional catheterization labs as at October, 2025.

Nigeria is the most populous African country with about 213 million people. It is estimated that about 10 percent of deaths in SSA is attributed to cardiovascular disease (12, 21). Despite this number, there only about 24 centers with cardiac intervention facilities in Nigeria with just slightly higher number of interventional cardiologists serving a population of over 200 million people (12) (Figure 1). In Kenya, the reperfusion rates for STEMI patient undergoing PCI are about 13%. Uganda has a single cardiac catheterization laboratory which is mainly funded by international organization and philanthropists (14, 22). Between Nigeria, Senegal, Ghana, Côte d’Ivoire and Cameroon there is an appalling total of 12 centres with facilities for PCI (23). Many countries in SSA still lack the capacity to manage cardiovascular diseases without cardiac catheterization laboratories or even trained cardiologists (24). Only few countries in SSA such as South Africa, Nigeria, and Kenya still have some capacity for managing interventional cardiology cases such as PCI (25). However, most of these centers are in the cities further widening the gap of healthcare accessibility between the rich and the poor. It is estimated that an average distance from a PCI facility in SSA is about 123.6 km which is about 100 min drive (26). Most of the facilities are in private hospitals where services are more expensive. In addition to the challenges, there is significant inequity in wealth distribution, which in turn affects health systems and facilities (27). This means that, even those whose might have access to nearby interventions may struggle with the economic burden and vice versa. In addition, limited availability of consumables, like stents and catheters, further impede the delivery of timely interventions (11).

## 4 Health workforce

The shortage of trained interventional cardiologists in SSA requires urgent attention. In SSA, there is about one cardiologist for every 600,000 individuals, while in the United States, there is roughly one cardiologist for every 13,000 people (28, 29). And there are fewer cardiologists who are skilled in interventional procedures. There have been efforts over the years to close this gap. For instance, in Nigeria, the field of interventional cardiology was started by foreign trained interventionists who come for regular cardiac missions, some which later established centers locally. From 2013, there was a cardiovascular education foundation group that began yearly skill trainings through seminars and practical skills transfer to young fellows and early career cardiologists. This stimulated a lot of growth in the manpower and availability as many young cardiologists developed interest in the field. Many of them also travelled for further training in high volume centers like in India. Unfortunately, most of these skilled cardiologists may not have a formal certification as interventional cardiologists despite having the needed skills. The scarcity of specialized training programs within Nigeria further exacerbates the shortage of interventional cardiologists. This lack of local training necessitates reliance on foreign training, which is often expensive and inaccessible to many. Furthermore, management guidelines that are based on best practices in resource-endowed nations may not be implementable because of delayed presentation and deficits in health care infrastructure (10). The combined lack of skilled manpower, economic disadvantage, and even knowledge about the range of services in interventional cardiology, contribute to the ever-growing morbidity and mortality in otherwise salvageable cardiovascular conditions (30).

## 5 Procedural demand and capacity

Despite the established high burden of cardiovascular disease and the need for cardiac intervention, the number of procedures performed per year in most of the centers in SSA is still low. The occasional increase in the number of procedures is mainly due cycle of missions when there is marked reduction in the cost of procedures. With only about 350 coronary interventions per year, Nigeria greatly lags behind compared to global standards. The Abidjan Heart Institute in Cote d’Ivoire performed only 166 PCI in a decade despite being the only center offering interventional cardiology 24 h in a day and seven days in a week in the country (17). In contrast, European countries perform thousands of percutaneous coronary interventions (PCIs) per million population annually (12, 30–32). For instance, Austria with a population of about 9 million people had 34 centers in 2017 and performed over 24,000 PCIs in the year (33). Moreover, the availability of advanced interventional procedures, such as transcatheter aortic valve replacement (TAVR) and complex structural heart interventions, is severely limited (29). Many patients requiring these procedures are forced to seek care abroad, incurring substantial costs and logistical challenges (30). Moreover, the procedures are mainly financed from out-of-pocket payment which significantly reduces the accessibility. Insurance cover and occasional government sponsorship account for only small proportion of cases.

These challenges underscore the systemic issues in cardiovascular disease care and the dire need for collaboration between interdisciplinary bodies to lend a hand in providing interventional services.

## 6 Practical recommendations

### 6.1 Strengthen collaboration and advocacy as a route to improvement: Nigerian cardiac missions as case study

Global health collaboration helps bridge skill gap in medical specialty training (34). International and local collaboration between centers is a strategic step towards increasing access to interventional cardiology by bridging gaps in skills, equipment, and training capacity. Centers in Nigeria could collaborate with international groups, organization or institutions who are skilled in interventional cardiology procedures to collectively bridge the skill gap. Collaboration could also be in form of establishment of a satellite branch of interventional cardiology center in sub-Saharan African country which could serve as a training center for Cardiologists who are interested in sub-specializing in interventional cardiology. For instance, an exchange program was organized between University Teaching Hospitals (UTH) of Lusaka, Zambia and the University of Maryland, Baltimore (UMB) for training physicians in infectious disease (35). This program facilitated transfer of skills and developing local training capacity of African professionals. Other potential collaboration opportunities include bilateral agreement between government, incentives, clinical trial research collaboration, and business partnership.

An example of the successful collaboration in Nigeria is the Cardiac Mission at the Lagos State University Teaching Hospital in collaboration with the cardiovascular education foundation, an international non-profit organization in the United States. The collaboration reduced the cost of interventional cardiology procedure significantly through the contribution of skilled manpower and donation of equipment by the NGO. The Cardiac Mission has supported over 500 patients within four years (36).

Beyond Nigeria, the Africa-Pace Program was another remarkable model which operated across 14 countries from 1996 to 2019 and implanted 542 permanent pacemakers. 11 out of these 14 countries had their first pacemaker through the program (19). Besides, it resulted in transfer of skills – local teams progressed from performing only 3% of implantation in 1996 to 98% by 2018 (36). Also, Sustain Health Development in Africa (SHARE) Program and Cameroon's Shisong Cardia Center demonstrates the impact of global collaborations by establishing fellowship programs and permanent facilities for capacity building in interventional cardiology and other advanced cardiac procedures.

Cardiac missions across West African countries including Ghana, Senegal, Guinea and Mali and Eastern African countries like Uganda, Kenya and Tanzania have successfully delivered interventional cardiology procedures through collaborative frameworks (19, 36).

### 6.2 Health investment through partnerships and endowment

Establishing cardiac catheterization laboratory (Cath lab) for interventional cardiology procedures is expensive. However, investment into this field would yield a long-term return on investment. With the aging population and growing cardiovascular diseases in the region, the demand for interventional cardiac procedures is increasing. Also, establishment of more Cath labs will provide access to services locally and reduce medical tourism. Besides, it will position African centers more competitive globally and drive international patients seeking quality care at lower cost and shorter waiting time compared to developed nations like the United Kingdom or United States. Public-private-partnership (PPP) provides opportunity for private organization to leverage public infrastructure and resources to establish interventional cardiology centers that is affordable and sustainable. For instance, partnership between FIRST E&P, a petroleum company with Healthy Heart Foundation and First Cardiology Consultants in Nigeria provides accessibility of interventional cardiology to low-income patients. Similarly, Cameroon's Shisong Cardiac Centre, supported by international partners serve as the only private center in Central Africa with facility for interventional cardiology (19).

Moreover, there is need to advocate for special funding dedicated to cardiovascular diseases (CVDs) by government and international funding organizations similar to the Global Funds for TB, malaria and HIV. This strategy has helped reduced the burden of major infectious diseases in LMICs over the last decades and should be replicated in tackling common non-infectious diseases especially CVD. This will promote global health equity and reduce morbidity and mortality from these diseases. Additionally, there is need to emphasize double burden of disease facing the country's healthcare. Government and policymakers need to direct resources to tackle both communicable and non-communicable diseases. African Union should support establishing regional Cath lab in each region and subsequently scaling to ensure all member states have at least one functioning center with 24/7 service.

Innovative financing strategies are required to sustain and increase access to interventional procedures in low-resource settings. There is need for insurance, and earmarking policy to generate funding for healthcare. For instance, earmarked tax on products that increase cardiovascular risks such as soft drinks, alcohol, fast foods, and cigarette could generate pool of fund to subsidize treatment of CVD diseases in government established Cath labs. This funding strategy has been proposed by the World Health Organization and implemented in some countries (37, 38). For instance, an earmarked tax policy on tobacco and alcohol implemented in the Philippines raised over USD 1.2 billion in the first year and enabled enrolment of about 45 million Filipinos in the National Health Insurance Programme (39). Similarly, in South Africa implemented the Sugar-Sweetened Beverage (SSB) tax and raised over USD 340 million over first two fiscal years (40). Although the revenue was not earmarked for healthcare, it showed the potential of raising funds through this model in Africa. In practice, a fully functional catheterization lab involves expenditure ranging USD 1 million to USD3 million per lab. Hence, about 5% to 10% ring-fenced allocation from the revenue generated from SSB tax annually can fund the establishment and maintenance of cath lab in public health facilities across the country. However, in the long term, revenue from earmarked products may reduce generally and affect the fund generated from their taxation.

Lastly, crowdfunding for patients in need of intervention can be championed by non-governmental organizations, groups or youth networks to mobilize funding to complement systemic funding approaches.

### 6.3 Awareness and capacity building for sustainability

Sustainable and equitable expansion of interventional cardiology across SSA requires strategic investments in awareness and capacity building among early career physicians including medical students. This will spur interests of young medical professionals in pursuing careers in the field addressing the severe workforce shortage. Opportunities in form of exchange programs, externship, and observership at centers offering interventional cardiology should be provided for medical students and resident doctors with budding interest in the specialty. Besides, cardiology organizations and professional bodies should organize webinars, seminars and workshops to discuss opportunities available for interventional cardiology training within and outside the region. Sponsorship programs for young health professionals to attend conferences and specialty training should be established by professional bodies of cardiologists. There is need for mentorship programs and career counseling for young medical doctors need to be institutionalized to guide young medical doctors through available pathways in interventional cardiology. For instance pairing early-career physicians with established interventional cardiologist creates sustainable knowledge transfer while providing insights into career development, research opportunities and clinical practice establishment across SSA.

## 7 Implication

Attaining equity in interventional cardiology across SSA aligns with the universal health coverage agenda by the WHO. CVDs account for a significant cause of premature mortality in SSA and without integration of interventional services in health facilities for equitable access, UHC framework will remain structurally deficient. Scaling up of interventional cardiology is beyond establishment of cath labs. It requires healthcare system strengthening such as improved referral networks, emergency response infrastructure, critical care capacity and good supply chain system for consumables. Institutionalising the training pathway within medical colleges, establishing accredited simulation centres, and creating mentorship framework will create sustainable pipeline for specialists trained within SSA. Ultimately, these will help reduce the disparity in the outcome of cardiovascular diseases across geographical and socio-economic status. It will contribute to early diagnosis and management of common cardiovascular diseases such as coronary artery diseases, peripheral vascular diseases and acute coronary syndrome. Hence, reducing morbidity and mortality from CVDs resulting in prolong life expectancy. Besides, it will motivate and initiate strategies to address other health challenges, thereby strengthening the Africa's healthcare system.

## 8 Conclusion

In conclusion, disparities in interventional cardiology access across SSA is a product of systemic underinvestment and inadequate prioritization of CVDs. Interventional cardiology is rapidly progressing and defines the future of definitive care for many cardiovascular diseases. There is need for unified commitment to key priority areas highlighted in this paper, that is, collaboration, investment and capacity building. Providing equitable access to interventional cardiology is a global health priority. This is achievable through collective resolution between the government and key stakeholders.

## Data Availability

The original contributions presented in the study are included in the article/Supplementary Material, further inquiries can be directed to the corresponding author.
